# Contrasting diversity patterns of breeding Anatidae in the Northern and Southern Hemispheres

**DOI:** 10.1002/ece3.5540

**Published:** 2019-08-15

**Authors:** Qing Zeng, Julian Reid, Neil Saintilan, Matthew J. Colloff, Guangchun Lei, Li Wen

**Affiliations:** ^1^ School of Nature Conservation Beijing Forestry University Beijing China; ^2^ Fenner School of Environment and Society Australian National University Canberra ACT Australia; ^3^ Department of Environmental Sciences Macquarie University Sydney NSW Australia; ^4^ Environment Energy and Science NSW Department of Planning, Industry and Environment Sydney NSW Australia

**Keywords:** divergence, null model, phylogenetic and functional diversity, productivity seasonality, random forest model, standard effect size, taxonomic

## Abstract

For sustaining ecosystem functions and services, environmental conservation strategies increasingly target to maintain the multiple facets of biodiversity, such as functional diversity (FD) and phylogenetic diversity (PD), not just taxonomic diversity (TD). However, spatial mismatches among these components of biodiversity can impose challenges for conservation decisions. Hence, understanding the drivers of biodiversity is critical. Here, we investigated the global distribution patterns of TD, FD, and PD of breeding Anatidae. Using null models, we clarified the relative importance of mechanisms that influence Anatidae community. We also developed random forest models to evaluate the effects of environmental variables on the Anatidae TD, FD, and PD. Our results showed that geographical variation in Anatidae diversity is hemispheric rather than latitudinal. In the species‐rich Northern Hemisphere (NH), the three diversity indices decreased with latitude within the tropical zone of the NH, but increased in the temperate zone reaching a peak at 44.5–70.0°N, where functional and phylogenetic clustering was a predominant feature. In the Southern Hemisphere (SH), Anatidae diversity increased poleward and a tendency to overdispersion was common. In NH, productivity seasonality and temperature in the coldest quarter were the most important variables. Productivity seasonality was also the most influential predictor of SH Anatidae diversity, along with peak productivity. These findings suggested that seasonality and productivity, both consistent with the energy‐diversity hypothesis, interact with the varying histories to shape the contrasting hemispheric patterns of Anatidae diversity. Phylogenetic diversity (PD) and FD underdispersion, widespread across the species‐rich, seasonally productive mid‐to‐high latitudes of the NH, reflects a rapid evolutionary radiation and resorting associated with Pleistocene cycles of glaciation. The SH continents (and southern Asia) are characterized by a widespread tendency toward PD and FD overdispersion, with their generally species‐poor communities comprising proportionately more older lineages in thermally more stable but less predictably productive environments.

## 1 INTRODUCTION

Rapid global biodiversity loss, driven primarily by anthropogenic disturbance to ecosystems (Pimm et al., 2014), has prompted exploration of spatial and temporal changes in biodiversity (Jarzyna & Jetz, 2017). Many such studies focus on taxonomic diversity (TD), which is often measured as species richness, that is, the total number of species in a community. Historically, species richness has been used as a major criterion for determining conservation priorities (Van Jaarsveld et al., 1998). However, the biodiversity concept includes many other aspects of biological variation, including genetic, functional, phenotypic, and phylogenetic variability, and the overlap of the priority regions across these diversity dimensions can be limited (Brum et al., 2017) Therefore, prioritizing conservation of hotspots of species richness can lead to loss of other facets of biodiversity (Veach, Minin, Pouzols, & Moilanen, 2017) in part because knowledge of species richness is insufficient to understand processes of species coexistence, community structure, and ecological function (Cadotte, Carscadden, & Mirotchnick, 2011; Pollock, Thuiller, & Jetz, 2017). This has led to the advocacy of accounting for multiple dimensions of biodiversity including phylogenetic diversity (PD) and functional diversity (FD), in making conservation decisions (e.g., Brum et al., 2017).

Phylogenetic diversity has been measured using phylogenetic relationships among taxa reflecting the evolutionary history of a community (Faith, 1992). FD reflects the diversity of ecological functions and has been conceptualized as how species are distributed in multidimensional niche space defined by functional traits, reflecting diversity of ecological functions (Petchey & Gaston, 2002). Integrated measurement of TD, PD, and FD can provide a comprehensive representation of biodiversity patterns and ecological functions (Calba, Maris, & Devictor, 2014; Pollock et al., 2017). This approach has been applied to the mapping of global biodiversity patterns of certain taxa (Safi et al., 2011), elucidating linkages between biodiversity and ecosystem functions and services (Cadotte, Dinnage, & Tilman, 2012; Flynn et al., 2009; Jarzyna & Jetz, 2017) for conservation policy (Pollock et al., 2017), and to provide insights on how ecological communities are shaped by evolutionary history and the environment (Calba et al., 2014; Jarzyna & Jetz, 2017; Ricklefs, 2007). For example, a community with high PD relative to TD indicates phylogenetic overdispersion; the community consists of mostly unrelated lineages, perhaps due to competitive exclusion as a long history of competitive interactions can cause evolutionary divergence in species niches (Violle, Nemergut, Pu, & Jiang, 2011). The “competition‐relatedness hypothesis” (Cahill, Kembel, Lamb, & Keddy, 2008) echoes Darwin's theory on the relationship between niche similarity and competition (Darwin, 1859). On the other hand, a community with low PD relative to TD indicates phylogenetic clustering; the community consists of closely related species that share similar physiological limitations and exhibit evolutionary niche conservatism, at least in terms of broad morphological and habitat similarities. Environmental filtering may promote the coexistence of closely related species (Webb, Ackerly, McPeek, & Donoghue, 2002). Similarly, niche filtering and partitioning can also lead to FD being higher (clustering) or lower (overdispersion) than expected in relation to TD (Maire, Grenouillet, Brosse, & Villéger, 2015; Safi et al., 2011).

Distribution patterns of TD, FD, and PD may not be spatially consistent (Brum et al., 2017; Devictor et al., 2010; Voskamp, Baker, Stephens, Valdes, & Willis, 2017), and their environmental determinants can differ as well (Chapman, Tobias, Edwards, & Davies, 2018). Sustaining the multifacet of biodiversity hence requires a detailed scientific understanding of the drivers of the variation in the biodiversity. Nevertheless, few studied have explored the patterns of TD, FD, and PD distribution at global scale, in particular, the spatial mismatches among these dimensions across the globe and its environmental determinants are rarely investigated.

Birds are well suited to global biodiversity studies because detailed phylogenetic and trait data are available (Jetz, Thomas, Joy, Hartmann, & Mooers, 2012; Wilman et al., 2014). The global pattern of bird TD follows the typical latitudinal gradient, increasing from the poles to the tropics (Jetz et al., 2012). However, nonpasserines, of which nearly half are waterbirds, have a peak diversification rate in mid‐to‐high latitudes of the Northern Hemisphere (Jetz et al., 2012, figure 3 therein). Because high diversification rates are often associated with high species riches (Wiens, 2007), the breeding waterfowl shows a peak at 45°N (Dalby, McGill, Fox, & Svenning, 2014). The reasons for this discrepancy are poorly known and warrant closer investigation (Dalby et al., 2014).

In this study, we use data on species distributions, traits, and phylogeny to examine global biodiversity patterns (TD, PD, and FD) of ducks, geese, and swans (Anatidae)—the largest family of waterbirds, but one not well studied from a global biodiversity perspective. Moreover, most species of Anatidae have broad distribution, covering large range of climatic conditions, thus are suitable macroecological study. We aim to understand and compare the processes shaping the patterns of global TD, PD, and FD of breeding Anatidae. We hypothesize that (a) global patterns of TD, FD, and PD are shaped by the prevailing environmental conditions predicted by the energy‐diversity hypothesis; (b) there are localized spatial mismatches among TD, FD, and PD; and (c) the divergency is caused by the different responses to environmental factors, which can be partly explained by evolutionary history. Specifically, we have the following objectives:

1. Mapping the global distribution of TD, PD, and FD of breeding Anatidae. This is a simple task of updating the species richness map of Anseriformes (of which 97% are from the Anatidae family) by Dalby et al. (2014).
2. Exploring the geographical variations of PD and FD in relation to TD. Although PD and FD are typically correlated with TD (Rodrigues, Brooks, & Gaston, 2005), communities with the same richness may differ in phylogenetic relatedness because of differences in evolutionary history (Webb et al., 2002) as well as in their functional traits due to distinct environmental conditions (Stevens, Cox, Strauss, & Willig, 2003). We identify areas where PD and FD are significantly higher or lower than expected from TD, accommodating communities with high irreplaceability in terms of the uniqueness of evolutionary histories (PD) and ecological niches (FD).
3. Using random forest to model the effects of environmental variables, including climate, land production, and hydrogeography, on TD, FD, and PD, to explain the underlying processes determining the observed biodiversity patterns.

## 2 METHODS

Our overall approach was as follows: First, we produced global maps of TD, PD, and FD of Anatidae at 1° × 1° resolution. We refer the 1° × 1° cells as “communities.” Neutral distributions of PD and FD were simulated with null models that fixed the number of species in each community and the global probability of presence of the species. Simulated null distributions were used to calculate standardized effect size of PD and FD (FD_SES_ and PD_SES_). We then identified regions with significantly higher or lower PD and FD as an indicator of whether environmental filtering or contemporary (e.g., competition) or historical (vicariance) and regional processes might account for observed departures and differences (Ricklefs, 2007). Finally, using a random forest model, we assessed the effects of environmental variables (climate, primary productivity, and hydrogeography) on TD, PD, and FD.

### 2.1 Data sources

#### 2.1.1 Anatidae distribution maps

We used distribution maps of species of Anatidae from BirdLife International (http://datazone.birdlife.org/) that included the categories “native resident,” “native breeding,” “season uncertain,” “re‐introduced,” “possibly extant” and excluded the categories “passage,” “native non‐breeding,” “introduced,” “origin uncertain,” “extinct” and “possibly extinct.” We acknowledge that there is great difference in waterbird distribution between breeding and nonbreeding seasons (Dalby et al., 2014). While both breeding and nonbreeding grounds are important for sustaining waterfowl populations, we focus on breeding season distribution patterns because of the direct relationships between waterfowl reproduction and breeding season conditions, particularly peak productivity (Baldwin et al., 2013; Maher & Carpenter, 1984). To derive species composition for each 1° square, we projected each species' range into a global equal area Mollweide projection (WGS84 datum) using ArcGIS (Version 10.4; ESRI inc.) and overlapped the distribution maps with a global land raster of 1° × 1° cells. The 1° global land raster was produced by aggregating the 3 arc‐second (∼90 m) spatial resolution “hole‐filled” SRTM (Shuttle Radar Topography Mission) dataset obtained from http://srtm.csi.cgiar.org/. A community was considered occupied by a species where polygon overlapped any part of a grid. The final species occurrence matrix was for 14,366 communities and 153 species.

#### 2.1.2 Traits

Functional traits relate to measurable features of an organism that determine its habitat requirements, ecological niche, functional role within an ecosystem, performance, and fitness (Cadotte et al., 2011, 2012). We used 14 traits belonging to four categories relating to fitness and resource use (Petchey, Evans, Fishburn, & Gaston, 2007) in order to quantify FD of Anatidae (Table 1). FD metrics were based on those of Petchey et al. (2007), subsequently used by Bregman, Sekercioglu, and Tobias (2014).

**Table 1:** The four trait types and 14 traits used to characterize functional diversity of the Anatidae

Trait type	Trait	Scale	Data source
Resource quantity	1. Body mass (g)	Continuous	Planet of Birds^a^
	2. Generation time (years)	Continuous	BirdLife International^b^
	3. Clutch size	Continuous	
	4. Distribution area (km^2^)	Continuous	
	5. Incubation duration (days)	Continuous	
Components of diet^c^	6. Invertebrates	Percentage	Wilman et al. (2014)
	7. Vertebrates	Percentage	
	8. Fish	Percentage	
	9. Plants	Percentage	
Main foraging substrate^c^	10. Water	Percentage	Wilman et al. (2014)
	11. Riparian	Percentage	
	12. Ground	Percentage	
Migratory status	13. Migratory	Binary	Planet of Birds^a^
	14. Wingspan (cm)	Continuous	

Note

Missing data were filled using relevant literature (e.g., Carboneras, 2018).

a URL: http://www.planetofbirds.com/ (accessed 23 June 2017).

b URL: http://datazone.birdlife.org/ (accessed 28 June 2017).

c Estimated percentage, total summed to 100.

#### 2.1.3 Phylogenetic diversity

From the website *A Global Phylogeny of Birds* (http://birdtree.org/), we generated a random subset of 100 phylogenetic trees for the 153 species, based on Hackett et al. (2008) (Figure S1). Methods to construct trees were described by Jetz et al. (2012). For each of the 100 phylogenetic trees, we calculated the PD index: the sum of the tree branch lengths connecting all species in a community (Faith, 1992). For each grid, the final PD index is the average of the 100 PD values.

#### 2.1.4 Functional diversity

Using the species‐by‐trait matrix (Table 1), we calculated the similarity coefficient of Gower (1971), converted it to a dissimilarity distance matrix (*D* = 1−*S*), and subjected it to hierarchical cluster analysis using the UPGMA method (Petchey & Gaston, 2002) to create a dendrogram (Figure S2). We calculated the FD index of Petchey and Gaston (2002) for each grid cell. This index is one of the most commonly used, enables comparison of our results with other studies. It does not require abundance data and performs well at predicting ecosystem functioning (Flynn et al., 2009).

#### 2.1.5 Environmental predictor variables

We used 52 variables to describe the spatial and temporal variation in environmental conditions among the 1° cell (Table S1).

##### Climate

We used the updated 30 s WorldClim data (http://worldclim.org/version2) to calculate the mean and within‐cell heterogeneity of the 19 Bioclimatic variables for each cell. The bioclimatic variables are derived from monthly temperature and precipitation values, presenting a dataset describing the mean, seasonality, trend, and extreme climatic conditions. They are widely used to describe species' ecological niches (Hijmans, Cameron, Parra, Jones, & Jarvis, 2005).

##### Productivity

We used NDVI (normalized difference of vegetation index) and actual evapotranspiration (AET) as indicators of productivity. For NDVI, we obtained 1 km × 1 km resolution, 10‐day composites of NDVI for the period 2000–2009 (Copernicus Global Land Service; http://land.copernicus.eu/global/). Using the 10‐year NDVI time series, we extracted four variables to describe the spatial and temporal variations of productivity: (a) Peak growing season productivity (mn.NDVI) was defined as the 95th percentile NDVI averaged over the 1° × 1° cell. We used the 95th percentile to avoid spuriously high NDVI values (Tuanmu & Jetz, 2015); (b) NDVI seasonality (season.NDVI) was defined as the mean of the 10 annual standard deviations of the 36 NDVI values, aggregated to 1° resolution; (c) interannual variation of productivity (annvar.NDVI) was computed as the standard deviation of the 10 yearly 95th percentile NDVI maps, aggregated to 1° resolution; (d) spatial heterogeneity of productivity (het.NDVI) was calculated as the standard deviation of a moving 3 × 3 window (i.e., the focal cell and its eight neighbors), averaged over the 1° × 1° cell. Using data sourced from the Global Soil Water Balance Geospatial Database (http://www.cgiar-csi.org), we also calculated mean, seasonality, and spatial heterogeneity of AET.

##### Hydrogeomorphology

Access to freshwater is essential for Anatidae feeding and breeding. We quantified lakes, reservoirs, rivers, and wetlands in each community using three data layers from the Global Lakes and Wetlands Database (GLWD, https://www.worldwildlife.org/pages/global-lakes-and-wetlands-database): GLWD‐1 for large lakes (≥50 km^2^) and reservoirs (storage capacity ≥0.5 km^3^), GLWD‐2 for permanent water >0.1 km^2^ not included in GLWD‐1, and GLWD‐3 for lakes, reservoirs, rivers, and different types of wetland. We constructed raster datasets for: (a) distance to nearest permanent water body (Distance.w), computed as the shortest Euclidean distance to the edge of waterbody polygons (from GLWD‐2) weighted by elevation; (b) density of lakes (Lake.den): the percentage of each community covered by large water bodies (from GLWD‐1); and (c) density of wetland (Wetland.den): the percentage of each community that is represented by wetland (from GLWD‐3). We also calculated the mean and heterogeneity of elevation using the 30‐s STRM digital elevation model (http://srtm.csi.cgiar.org). Finally, we obtained two more variables for available water: the annual reliable runoff (m^3^/year) and percentage irrigated area in each community (GWSP Digital Water Atlas, http://atlas.gwsp.org). We used R version 3.3.3 (R Development Core Team, 2016) for the manipulation and calculation of spatial data.

### 2.2 Mapping faunal regions based on Anatidae composition

We used UPGMA clustering to classify the global land into faunal regions. The original global grids were reduced to 12,496 communities by removing two species with restricted (island) distributions and any community with less than four Anatidae species. UPGMA classification was performed by applying hierarchical clustering in the R base package “hclust()” to the Sørensen dissimilarity matrix, which was computed from the Anatidae community data using the “vegan” package (Oksanen et al., 2013).

The number of meaningful groups was visually assessed, by plotting the mean within‐groups dissimilarity against number of groups from one to 15 and by balancing the reduction in within‐group dissimilarity with an increasing number of small, trivial groups.

### 2.3 Exploring the geographical variations of Anatidae PD and FD in relation to TD

We used null models to determine whether observed global PD and FD distributions were significantly different from what was expected under the null hypothesis, that is, that Anatidae communities of each community consist of random draws of a fixed number of species from the global species pool (Villéger, Mason, & Mouillot, 2008). We created 999 null models by randomizing phylogenetic relatedness (for PD) and functional traits (for FD) of the species, that is, by creating random communities of the same TD through randomly re‐allocating species to the phylogenetic (and functional) tree tips. By randomizing the phylogenetic and functional trait data, the null models constrain the covariances and overall community patterns and are rendered less susceptible to biases in community data matrix randomizations (Swenson, 2014). With the random community PD and FD values, we also calculated a standard effect size (SES):

$$\text{SES}=\frac{\text{obs}-\text{mean}\left(\text{null}\right)}{\text{SD}\;\text{of}\;\text{null}}.$$

Positive SES values indicate higher phylogenetic (functional) diversity than expected in relation to the species pool, while negative SES values indicate lower diversity. We used the quantile score (i.e., where the observed value lands in the null distribution) as an estimate of *p*‐value to evaluate the significance of the divergence. If the quantile score is ≤25, the community has significantly lower PD (FD) than expected. If the quantile score is ≥975, the community has significantly higher PD (FD) than expected.

### 2.4 Modeling the impacts of environmental variables

Based on the faunal regions derived from UPGMA clustering (Figure 3), we separated the global TD, PD, and FD distributions into Northern Hemisphere (NH, including clusters 1–6, Figure 3) and Southern Hemisphere (SH, including clusters 7–12, Figure 3). The diversity indices showed strikingly different gradients between NH and SH, suggesting that environmental conditions might structure the bird community differently. Thus, we developed separate models for the two hemispheres to evaluate the environmental determinants. We analyzed TD, PD, and FD using random forest implemented in the R package “randomForestSRC” (Ishwaran & Kogalur, 2014). Random forest is a common machine learning method for classification, though less applied to regression (Oliveira, Oehler, San‐Miguel‐Ayanz, Camia, & Pereira, 2012), but with useful features: (a) Random forest is a nonparametric rule‐based algorithm which performs better than parametric methods for complex systems (Breiman, 2001); (b) it can deal with nonlinearity and interactions among predictors better than generalized linear and additive models (Oliveira et al., 2012); (c) the algorithm estimates the importance of a variable by determining the increase in prediction error when “out‐of‐bag” data for that variable is permuted while all other data are left unchanged (Grömping, 2009), which is of value because an objective of this study was to identify environmental variables that are most important in determining global patterns of Anatidae diversity.

Multicollinearity can bias estimates of the importance of predictor variables (Siroky, 2009). To limit this effect, we prescreened the 52 predictor variables using the variance inflation factor (VIF): Variables with VIF >10 were excluded (Fox & Monette, 1992) leaving 26 variables (Table S1) for random forest modeling. Moreover, as all three diversity indices showed strong spatial autocorrelation (Moran's *I* test, *p* values <.001), we included latitude and longitude of the center of each community into the random forest models (Wood, 2006). For each diversity index, we grew 1,000 trees, so the result was the mean of 1,000 predictions. In growing a tree, we used 11 predictor variables for each step of splitting, which was based on weighted mean‐squared error (Breiman, 2001).

## 3 RESULTS

### 3.1 Global pattern of TD, FD, and PD

**Figure 1:**
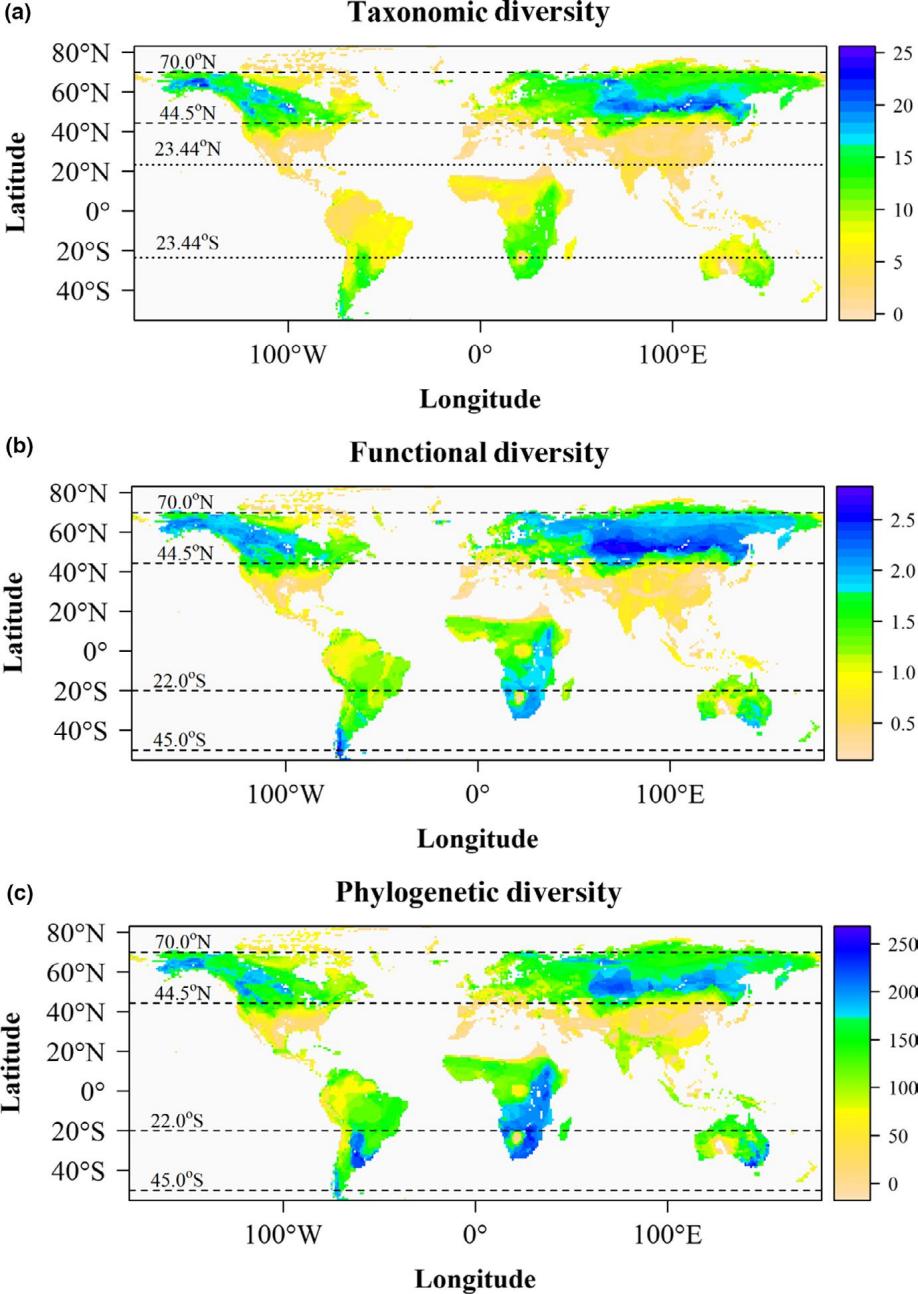
Global pattern of (a) taxonomic diversity (TD); (b) functional diversity (FD); and (c) phylogenetic (PD) diversity of breeding Anatidae. Large deserts (e.g., the Sahara) and water bodies (e.g., Lake Superior) had no occurrence of Anatidae. The tropics, showed as between the dotted lines in (a), have relatively low TD, FD, and PD except in the Upper Nile and Zambezi Basins in east and central Africa. TD is highest in the band between latitudes 44.5–70°N (dashed limes), and FD and PD are highest in bands between latitudes 44.5–70°N and 20–55°S (dashed lines)

There was a very similar spatial pattern for all three diversity indices (Figure 1): TD, FD, and PD are highly correlated (Pearson's *r* > .90 for all paired comparisons) as the indices are intrinsically related (Safi et al., 2011). The NH had greater TD than the SH; communities with ≥18 species were all located in the northern temperate and sub‐Arctic zones (Figure 1a). There was no increase in species richness toward the equator as found for plant and other animal taxa (Hillebrand, 2004; Lamanna et al., 2014). Species richness was low (<5 species per cell) in the tropics except in the Middle Nile and Zambezi basins, northern Australia and the northern Parana‐La Plata River basin of central South America (Figure 1a). In the Northern Hemisphere, there is an extensive band of high species richness over much of central and western North America and central and eastern Asia (between ca. 47 and 65°N). Within this band, regions of highest species richness (>20 spp.) were quite consistent and included the middle‐lower Yangtze Basin in China, the Yenisey, Baikal, Amur, Lenn, and Ob river basins in Siberia and Central Asia, and most of river basins of northwestern North America (Figure 1a).

In the NH, the pattern of FD was similar to that of TD, although the high FD band between 45 and 70°N was broader than that for TD (Figure 1b). Compared with TD, FD was relatively high in the SH, with high FD (>1.25; global mean 0.95) in Patagonia and the Murray–Darling Basin of eastern Australia. Most of southern Africa had moderately high FD except in its deserts.

The SH had higher PD than the NH (Figure 1c). The south and east coast of Australia and the Murray–Darling Basin, the Parana‐La Plata Basin of South America, the Zambezi and Okavango basin of southern in Africa had high PD (>200; global mean 123). In North America and across Eurasia, areas of higher PD generally corresponded with areas of maximum TD and FD, that is, within the 45–70°N band.

Despite spatial discrepancies between regions with high TD, FD, and PD, there was a consistent pattern for regions with low diversity (Figure 1): in deserts, high mountain regions (such as the Himalaya Plateau, Andes, and the Ethiopian Highlands), and oceanic islands (e.g., those in the western Pacific and Arctic Ocean).

### 3.2 Patterns of divergence of FD and PD from TD

Phylogenetic and functional clustering, indicated by the negative SES values, was generally observed in the Northern Hemisphere. In contrast, overdispersion, indicated by the positive SES values, mainly occurred in the SH (Figure S3).

**Figure 2:**
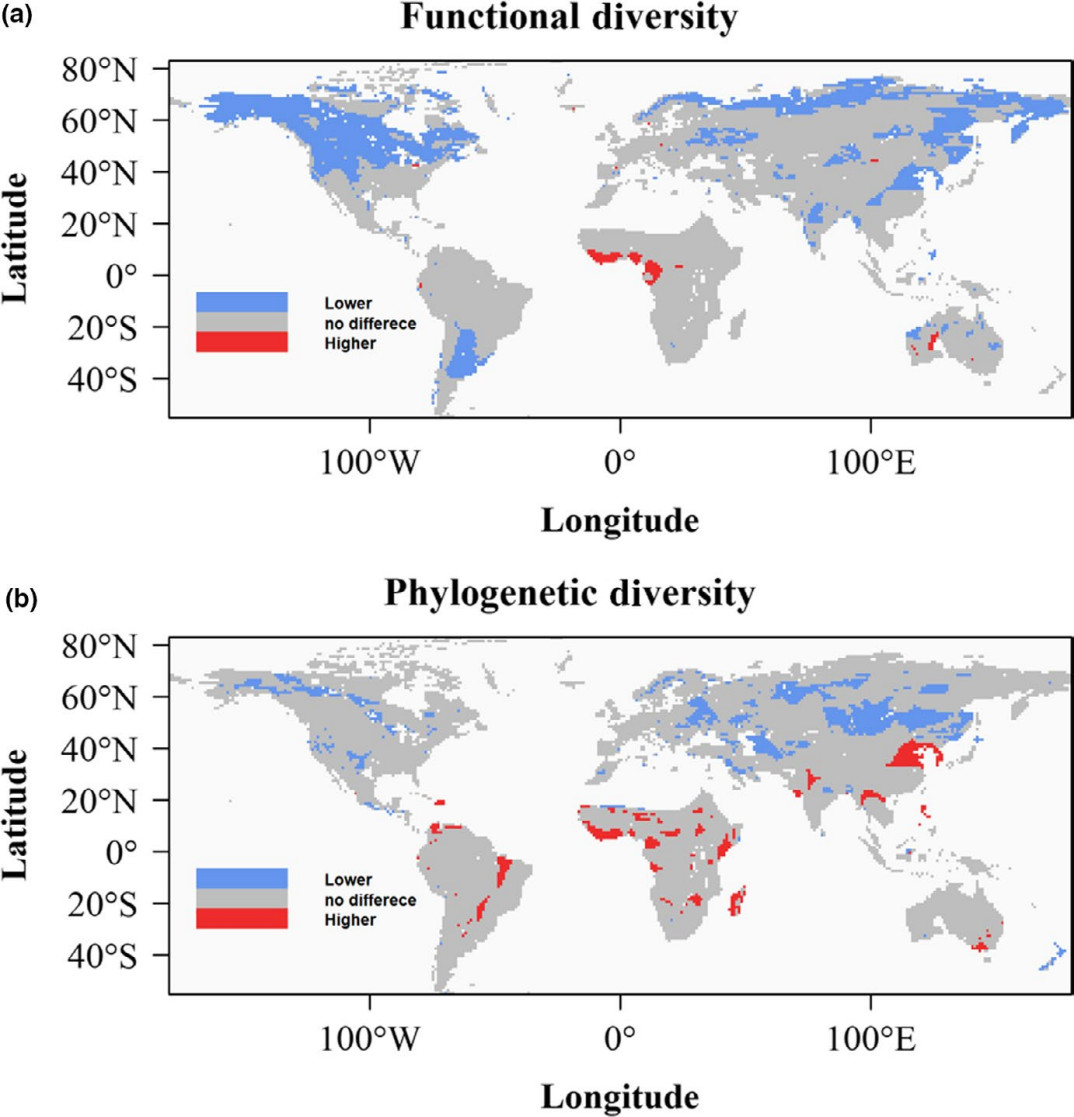
Maps showing regions where (a) functional diversity and (b) phylogenetic diversity of breeding Anatidae is significantly higher or lower than expected relative to observed taxonomic diversity based on 999 null model simulations

More than 20% of communities had significantly lower FD than predicted by the null model, and only 1% of communities had significantly higher FD than predicted (Figure 2a). Most of the functional clustering was in the Northern Hemisphere, covering the Arctic region and Canada, with patchy distribution across Eurasia, the Indian subcontinent, northern Australia, and the Parana‐La Plata Basin of South America. The Gulf of Guinea and West Africa was the main region with significantly overdispersed FD.

About 11% of communities had significantly lower PD than predicted (Figure 2b). Nearly, all these areas were in the NH. However, overdispersion of PD was more common than FD: 4.2% of communities had significantly higher PD than predicted, mostly within tropical and subtropical regions, with patchy distribution across the SH, especially sub‐Saharan Africa and southeast Australia. Despite some overlap between regions with significantly higher or lower FD and PD, generally there was little spatial concordance. One region, the Yellow Sea Basin (including the lower Yellow River, the lower Yangtze River and the Korean Peninsula), had clustered FD but overdispersed PD, indicating that the species of Anatidae of this region share many functional traits but are drawn from several independent phylogenetic lineages. In other words, they appear to demonstrate ecological convergence.

Patterns of significantly lower FD and PD in the species‐rich, northern temperate and sub‐Arctic regions of North America and Eurasia reveal the curious trend that much of the North American region has clustering of functional traits, while the central Eurasian region (the Mongolian plateau and parts of Siberia) showed phylogenetic clustering.

### 3.3 Faunal regions based on Anatidae composition

**Figure 3:**
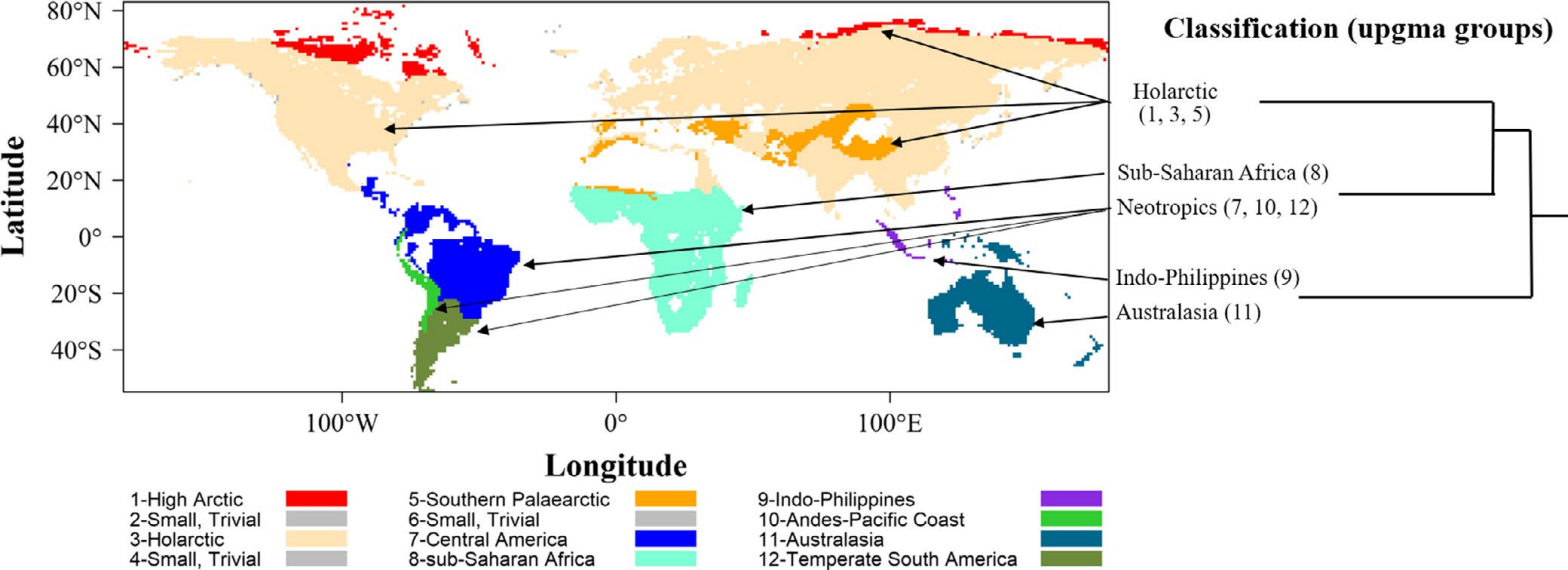
Map of faunal regions based on Anatidae composition of 1° cells. The simplified dendrogram of the UPGMA is presented in the right

Twelve groups were recognized of which three were trivial and peripherally distributed in the NH (Figure 3). The primary split in the dendrogram separated Australasia and adjacent southeast Asian archipelagos from the rest of the world, emphasizing the long period of geological and evolutionary isolation of the Australian plate and its terrestrial biota (Edwards & Boles, 2002).

The second split separated the Afrotropical and Neotropical realms from NH groups. At the 12‐group level, the major central American groups (including the Amazon Basin and north Atlantic coast) and single Afrotropical group were more similar in species composition than two smaller South American groups (Andes‐tropical and subtropical Pacific coast, and temperate South America, Figure 3).

Three major groups could be discerned in the NH (Figure 3): a general Holarctic group (North America + Eurasia), comprising 60% of all communities included in the analysis, a high Arctic group, and a southern Palaearctic group comprising species‐poor communities in the Mediterranean, Caspian and Black Seas and Himalayan region. Mean species richness in the main Holarctic group was 15.5 compared with 5.9 species in the southern Palaearctic group. Temperate South America had the second highest mean species richness per community (14.2).

### 3.4 Environmental determinants of TD, PD, and FD

**Figure 4:**
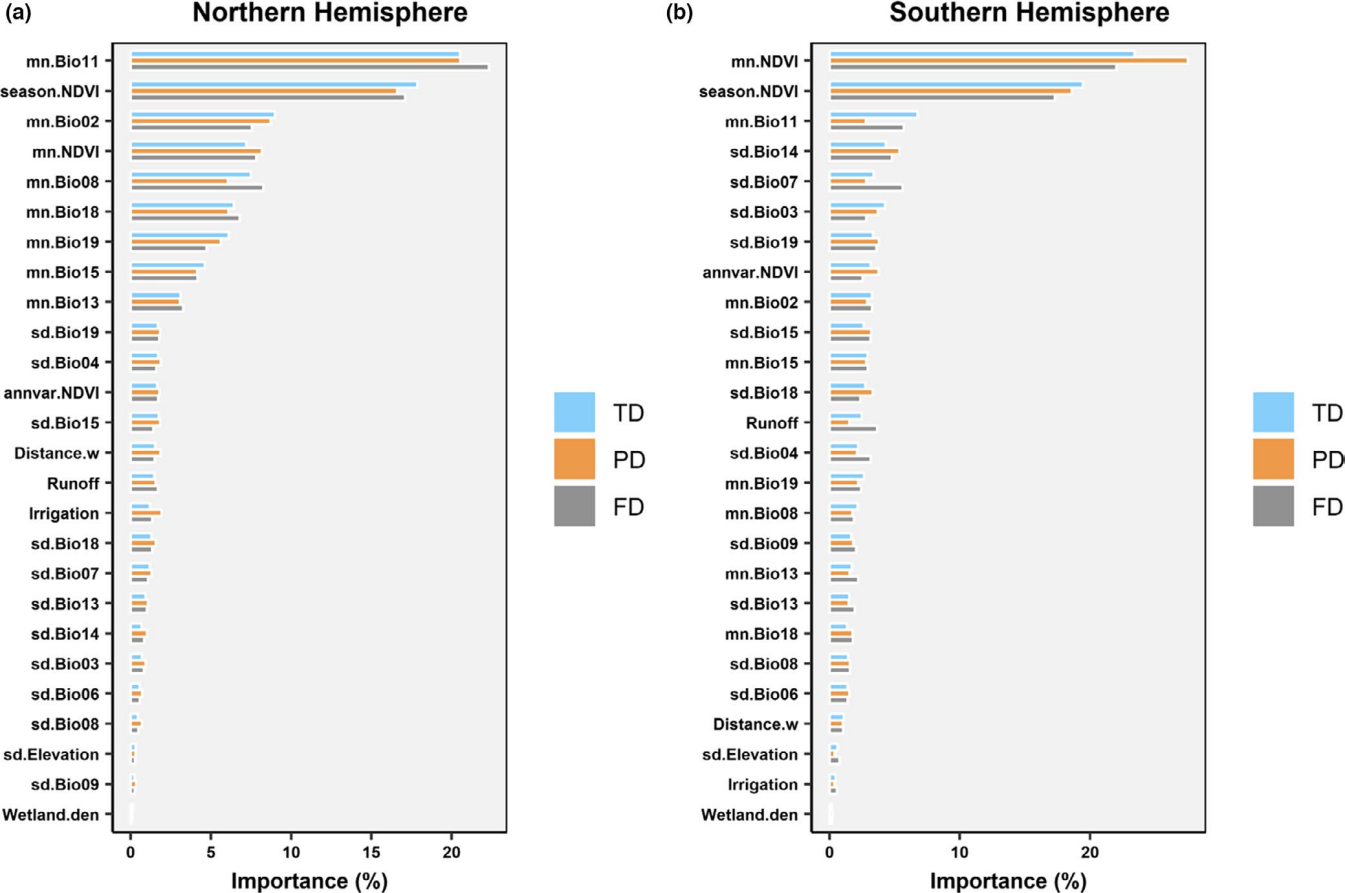
The relative importance of environmental predictor variables of diversity of breeding Anatidae in the Northern (a) and Southern Hemisphere (b), determined using random forest models. Scores of importance were scaled so that the total is 100. Latitude and longitude of each cell were included in the models to account for spatial autocorrelation and were ranked high (especially latitude), but were excluded from the graphs to allow for clearer illustration of the relative importance of the environmental predictors. mn.Bio01—mn.Bio19 and sd.Bio01—sd.Bio19 are the cell mean and cell standard deviation of the 19 bioclimatic variables by Hijmans et al. (2005). mn.NDVI, season.NDVI, and annvar.NDVI are mean peak, seasonality, and interannual variation of NDVI. Description and calculation of other variables can be found in Table S1

**Figure 5:**
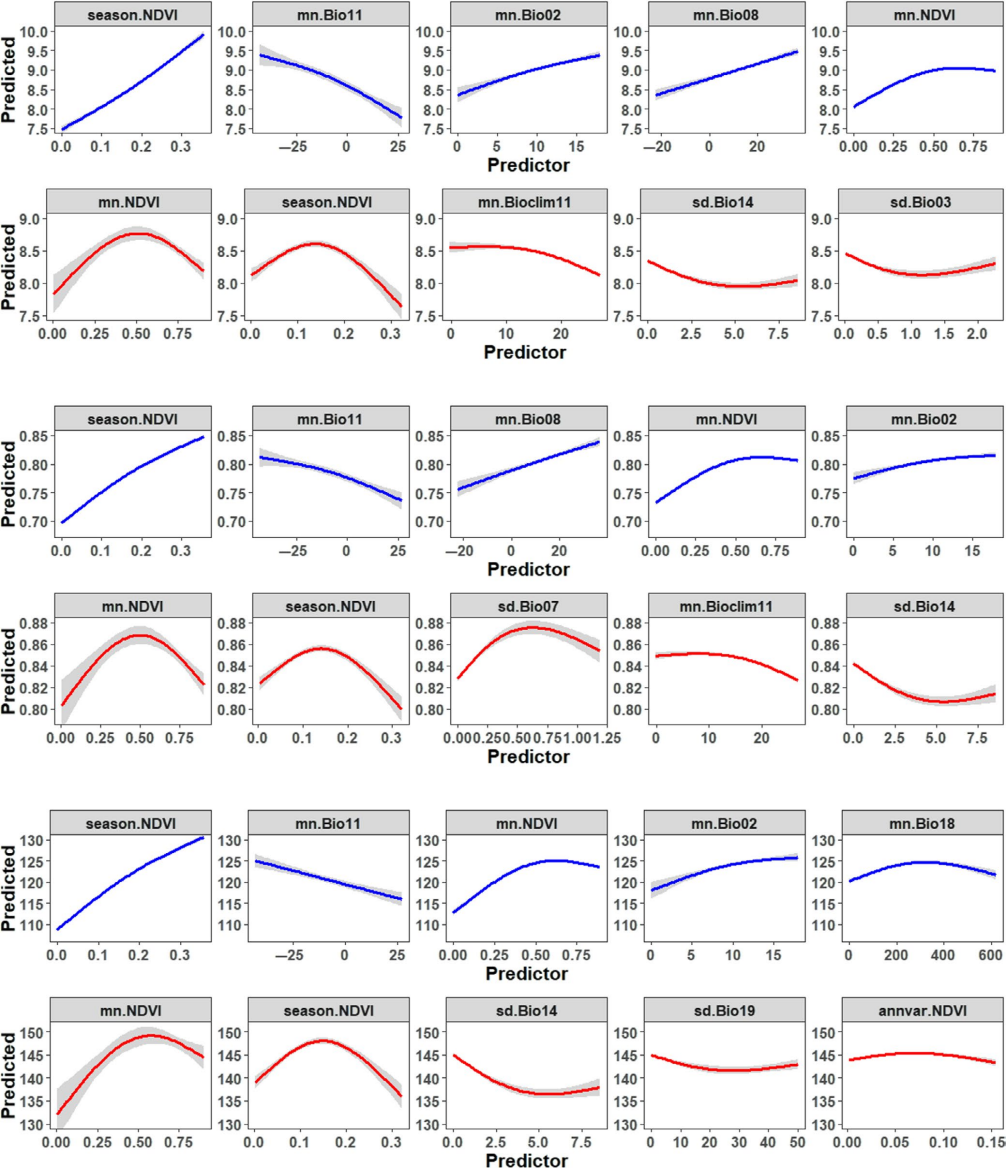
Partial response curves to the top five environmental predictor variables for taxonomic diversity (upper), functional diversity (middle), and phylogenetic diversity (lower) of breeding Anatidae. Line = partial value; gray shade = ±*SE*. For each panel, NH models are blue at top and SH are red at bottom. See Figure 4 for description of the environmental variables

The most influential environmental predictors (Figure 4) and response curves (Figure 5) were distinct for NH and SH.

#### 3.4.1 Northern Hemisphere

The random forest models performed well in predicting the distribution of the Anatidae diversity metrics in the NH (the percentage of variance explained was 93.36 and 93.12 and 90.65 for TD, FD, and PD, respectively). Although ranked slightly differently, the top predictors were the same for the three biodiversity metrics (Figure 4a). Two variables, that is, season.NDVI (productivity seasonality) and mn.Bio11 (mean temperature at the coldest quarter), were much more important for Anatidae diversity than other environmental predictors (Figure 4a). Many other variables describing the prevailing climatic conditions, including mean diurnal temperature range, mean temperature of the wettest quarter, precipitation seasonality, and total precipitation in the warmest quarter, were among the top predictors. In contrast, variables related to the spatial heterogeneity of climatic conditions were ranked low in predicting the biodiversity patterns, as were hydrogeomorphology variables.

We extracted the top five predictor variables based on minimal depth (Ishwaran & Kogalur, 2014) and used partial plots to examine their marginal effect on the diversity indices (Figure 5).

##### Productivity

For all three diversity indices, season.NDVI was among the most important environmental variables (Figure 4a). The partial response curves showed that Anatidae diversity increased with season.NDVI. The marginal effects of season.NDVI on TD were almost linear, but more nonlinear for FD and PD, that is, the rapid increase of diversity with season.NDVI leveled off at higher values (Figure 5). The mean land peak productivity (mn.NDVI) was also positively associated with Anatidae diversity, and the partial response curves were nonlinear and similar for all three indices: The diversity indices increased with mn.NDVI until about 0.5, after which the effect leveled off (Figure 5).

##### Climate

The most important climatic variable was the mean temperature of the coldest quarter (mn.Bio11, Figure 4a), and all three diversity metrics decreased with increasing mn.Bio11 (Figure 5). In contrast, the Anatidae diversity metrics increased with the mean temperature of the wettest quarter (mn.Bio08), which was also ranked high in predicting Anatidae diversity. Other climatic variables describing the interaction of temperature and precipitation, expressed as grid means, were also important determinants of Anatidae diversity (Figure 4a). Spatial heterogeneity measures of these Bioclimatic variables were of low importance.

#### 3.4.2 Southern Hemisphere

Compared with the NH random forest models, the SH models explained less percentage variance (81.98%, 77.57%, and 80.51%, for TD, FD, and PD, respectively). More importantly, the top environmental variables which contributed most to model performance showed considerable differences with those in the Northern Hemisphere. The top two most important variables, which were far more important than the others, were the same for TD, FD, and PD and related to productivity (mn. NDVI, the mean peak NDVI and season.NDVI, the seasonality in NDVI: Figure 4b). Other important predictor variables varied for the three metrics reflecting the greater divergence of FD (PD) from richness in the SH (Figure 3). As with the NH models, the hydrogeomorphology variables ranked low except runoff for FD (Figure 4b).

##### Productivity

The top two most important variables related to productivity (Figure 5). The partial response curves of mn.NDVI and season.NDVI were similar and strongly nonlinear for TD, FD, and PD (Figure 5). The unimodal curves were remarkably different compared with these of the NH models, which were monotonic. The effect of productivity on the three diversity indices was similar: the diversity indices peaked at NDVI ~0.50. The peak value for season.NDVI was ~0.15 (Figure 5).

##### Climate

Unlike the NH models, the set of climatic variables responsible for the diversity variations was different for TD, FD, and PD. Moreover, the variables describing spatial heterogeneity of climatic conditions were more important for the Anatidae biodiversity in Southern Hemisphere (Figure 4b).

For TD, mn.Bio11 was the most important climatic variable. The partial response curves showed a generally negative relationship between TD and mn.Bio11: The TD was relatively stable until temperature reached ~10°C, after which diversity decreased (Figure 5). Other important climatic variables for TD included the spatial heterogeneity of precipitation in the driest month (sd.Bio14) and isothermality (sd.Bio03). For FD, the most important climatic variables included the spatial heterogeneity of sd.Bio07 (temperature annual range), mn.Bio11, and sd.Bio14. For PD, the most important climatic variable was the spatial heterogeneity of sd.Bio14 and precipitation in the coldest quarter (sd.Bio19; Figure 5).

## 4 DISCUSSION

In this study, we mapped and examined the global distribution patterns of TD, FD, and PD of Anatidae, of which 30 species are threatened or endangered worldwide (Austin, Slattery, & Clark, 2014). Our results showed there were great similarities between the distribution patterns of FD and PD (Figure 1b,c) and how they respond to environmental gradients (Figure 5), which are consistent with the assumption that species traits reflect their shared evolutionary history (Cadotte et al., 2012). Traits are often highly conserved along evolutionary lineages, and sometimes, PD has been used as a proxy for functional composition in conservation decisions (Winter, Devictor, & Schweiger, 2013, but see Mazel et al., 2018 for other opinions). Duo to the analogous behaviors of PD and FD, our discussion will mainly focus on PD.

Our results revealed a prevailing pattern of clustering in FD and PD indices of breeding Anatidae, indicating niche filtering might be the dominant mechanism that shapes communities at global scale (Webb et al., 2002). These results support our first hypothesis. However, our null models revealed the striking difference in the divergence of FD and PD from TD between the SH and NH (Figure 3), confirmed our second hypothesis: While clustering effect was stronger and dominant in shaping Anatidae community in the NH, overdispersion was common in the SH. Finally, the random forest models indicated that all three biodiversity indices were strongly affected by productivity seasonality, confirming the finding of Dalby et al. (2014) in that temporal niche exploitation was the main underlying mechanism shaping waterfowl distribution at global scale. Moreover, our results showed that while climatic variables, especially those describing temperature seasonality and the interaction between temperature and precipitation, were important determinants in the NH, variables related to productivity were more decisive in the SH. In addition, the response of diversity indices to environmental gradients is strongly nonlinear in SH while more or less linear in NH (Figure 5). These results provide evidences partly supporting the third hypothesis.

### 4.1 Global distribution patterns of TD, FD, and PD

Geographically, the major differences in diversification rates in birds are hemispheric rather than latitudinal (Jetz et al., 2012). This may lead to the distinct hemispheric patterns in Anatidae diversity. In the NH, the PD and FD peaked at the band of 44.5–70.0°N (Figure 1) similar to Anseriformes richness as reported by Dalby et al. (2014). In the SH, Anatidae diversity generally increases with distance from the equator (Figure S4), which is opposite to the general latitudinal diversity gradient (Hillebrand, 2004).

There is a clear latitudinal divide at 20–30°N between regions of the world in which PD is less (NH) and greater (SH) than that expected from observed TD, except in China where this divide occurs at a higher latitude (50°N). Global patterns of speciation for all birds reflect our findings of global patterns of diversity for Anatidae, with lower rates in Australia, South‐East Asia, Africa, and Madagascar, and higher rates in North America, northern Asia, and southwest South America (Jetz et al., 2012). The rate of diversification among Anatidae is particularly high compared to other bird lineages, with relatively rapid speciation in the past 10 million years (Jetz et al., 2012). However, this diversification is geographically constrained, with a particularly high phylogenetic dispersal in the SH. Jetz et al. (2012) suggested that the low diversification rate on the SH continents is related to the filling of ecological niches by ancient clades in their region of origin (Ericson, 2012). The high PD of waterbirds compared to TD south of 40°N is attributable to the stability of populations of older lineages in continents long isolated and less subject to widespread glaciation: South America, Australia, and, to a lesser extent, Africa.

North of 40°N, where the breeding grounds and centers of diversity for waterbirds are concentrated, land masses have been subject to strong climatic fluctuations and periodic glaciation during the Pliocene and Pleistocene epochs, which suggests that the expansion, contraction, and altered location of wetland habitat has been a driver of the high rate of diversification, particularly over the past 10 million years (Jetz et al., 2012), and in particular, three phases of extreme diversification during the Pliocene–Pleistocene (Sun et al., 2017).

### 4.2 Environment drivers of diversity patterns

Although the number of environmental variables influencing Anatidae composition is potentially very large, our results indicated that a few key variables were sufficient to predict Anatidae distribution, might reflect the association among the environmental variables (Siroky, 2009). In the NH, out of the 26 variables we investigated, two (season.NDVI and mn.Bio11) are far more important than others (Figure 4). The main driver of diversity in the NH is seasonality in NDVI, highlighting the importance of the northern Tundra and subarctic region for periodically high productivity during the northern summer. Anatidae diversity in the NH increases with seasonality of NDVI, which follows the seasonal patterns of temperature and rainfall (Potter & Brooks, 1998) corresponding to high latitude continental climates. These locations are subject to high seasonality in productivity over annual cycles, allowing an influx of many closely related species of breeding waterbirds during the resource “boom” of the northern summer when there is little evidence of density dependence (Elmberg, Nummi, Poysa, & Sjoberg, 1994). Niches are unlikely to be tightly partitioned: Evolutionary radiation seems driven by the disruptive effects of glacial–interglacial cycles over the Pliocene and Pleistocene epochs, as described above. In addition, the partial response curve indicated that Anatidae diversity metrics are negatively correlated with the mean temperature of the coldest quarter (mn.Bio11, Figure 5), which generally decreases poleward. This negative relationship is seemingly opposite to the energy‐diversity hypothesis (Hurlbert, 2004). This disagreement might be due to the high component of migrants in the NH communities, and they are generally absent from their breeding grounds during the coldest quarter.

In the SH, given the absence of continental land masses in sub‐Antarctic latitudes, resource seasonality, including climate (especially temperature) and productivity (Figure S5), becomes less distinct. The lack of strong seasonal signals might lead the large proportion of nonmigratory birds in the SH communities (Dalby et al., 2014). Thus, the variables, which are related to the availability and temporal variation of resources such as peak productivity (mn.NDVI) and interannual variability (annvar.NDVI), become relatively more important for Anatidae diversity in the SH than in the NH (Figure 4b). These results support the energy‐diversity hypothesis (Hurlbert, 2004) better than the competitive exclusion principle (Cahill et al., 2008). Nevertheless, the high relative importance of productivity seasonality on Anatidae diversity in both hemispheres reflects the high capacity of Anatidae to exploit seasonal food resources via short‐ and long‐distance migration (migration as in Dalby et al., 2014; dispersal as in Wen, Saintilan, Reid, & Colloff, 2016). On the Australian continent, temporal resource availability is more stochastic, related to the periodic and unpredictable flooding of dryland river systems related to fluctuations in the Southern Oscillation Index, and the provisioning of food resources on floodplains during wet periods (Baldwin et al., 2013). Intermittently flooded wetlands are enormously productive during of major floods and support diverse communities of breeding Anatidae (Reid, Colloff, Arthur, & McGinness, 2013), comprising more than two thirds of the Australian Anseriformes such as Plumed Whistling‐Duck, Black Swan, and Grey Teal. Here too, a lack of density dependence may promote periodically high diversity in the absence of predator‐prey density dependence.

As most species of Anatidae prefer open low vegetation (Thompson, Arnold, & Amundson, 2014), the contribution of woody biomass to productivity might explain the distinct response curves of diversity indices to productivity and seasonality between NH and SH (Figure S5). In NH, areas of high peak productivity overlap with those of high seasonality, coincide with the temperate boreal forest (the Taiga), where tree biomass is generally below 65 tonnes per ha (Hengeveld et al., 2015). In SH, on contrast, the areas with high peak productivity (as well as relatively high seasonality, Figure S5) are big tropical forests (e.g., Congo Basin, Amazon) with woody biomass normally >100 tonnes per ha (Hengeveld et al., 2015).

In common with the NH, the deserts and mountain ranges of the SH present climatic and topographic impediments to formation of waterbird habitat. However, regions of high water availability and large wetlands are not necessarily associated with high TD, FD, or PD at the spatial scale of this study. Several large river basins such as the Amazon (including the Pantanal), the Nile, and the Congo maintain low TD and PD, in spite of extensive wetlands. These wetlands are located in lower latitude regions that have low seasonal variation in productivity (Figure S5). This finding might be surprising, given the importance of habitat area in controlling diversity of forest birds (Boecklen, 1986). However, our findings at global scale support the observations of Bethke and Nudds (1993) in Prairie ecosystems that the diversity of Anseriformes increases, rather than decreases, with environmental variability, which might lead to resource variability (Dalby et al., 2014). Large wetland systems in regions of limited climatic variability do not meet the conditions of intermediate disturbance associated with the maintenance of high diversity.

### 4.3 Conservation implication

Habitat availability for Anatidae in the NH is abundant, whereas in the SH habitat is resource‐limited and patchy (as evidenced by the map of global seasonality of land production, Figure S5). At the global scale, the implication for conservation is that in the SH priorities would focus on habitat conservation, as well as mitigation of populations threats such as hunting and harvesting, whereas in the NH, the emphasis would be on threats other than habitat availability. However, there are other important drivers other than biophysical variables. Amano et al. (2018) found the strongest predictor of waterbird abundance and of beneficial conservation outcomes was effective governance. The institutional and political stability of social‐ecological systems in which Anatidae and wetlands are prominent components represents a major determinant of effective conservation effort and adaptive management.

More importantly, this study raises the question of redundancy and resilience in species‐poor communities across the SH and tropical regions generally. Given the relatively high FD of these impoverished waterfowl assemblages, ecosystem services and functions would be lost were individual species to become extinct or undergo large range contractions, due to the absence of locally occurring, functionally similar, potential‐replacement species. The imperative to manage wetlands in these regions wisely and conserve all species is as great as ever.

## CONFLICT OF INTEREST

None declared.

## AUTHOR CONTRIBUTIONS

QZ, NS, and LW conceptualized the manuscript; all authors involved in methodology, validation, and writing—review and editing; LW and QZ involved in software; LW, JR, and QZ formally analyzed the manuscript; LW, JR, and MC involved in writing—original draft preparation; and LW involved in visualization.

## Data Availability

Table S2 “Species‐trait matrix,” Table S3 “Species‐community matrix” and Table S4 “Biodiversity indices—community—environmental variable matrix” can be accessed Dryad Digital Repository (https://doi.org/10.5061/dryad.g0j48c7). All other supporting materials (i.e., Table S1, Figures [Link], [Link], [Link], [Link], [Link]) can be found in the supplementary information.
