# Supplementary figures and images for: Contrasting diversity patterns of breeding Anatidae in the Northern and Southern Hemispheres
Source: Ecol Evol. 2019 Aug 15;9(17):9990–10003. doi: 10.1002/ece3.5540 (PMC6746110; doi:10.1002/ece3.5540)

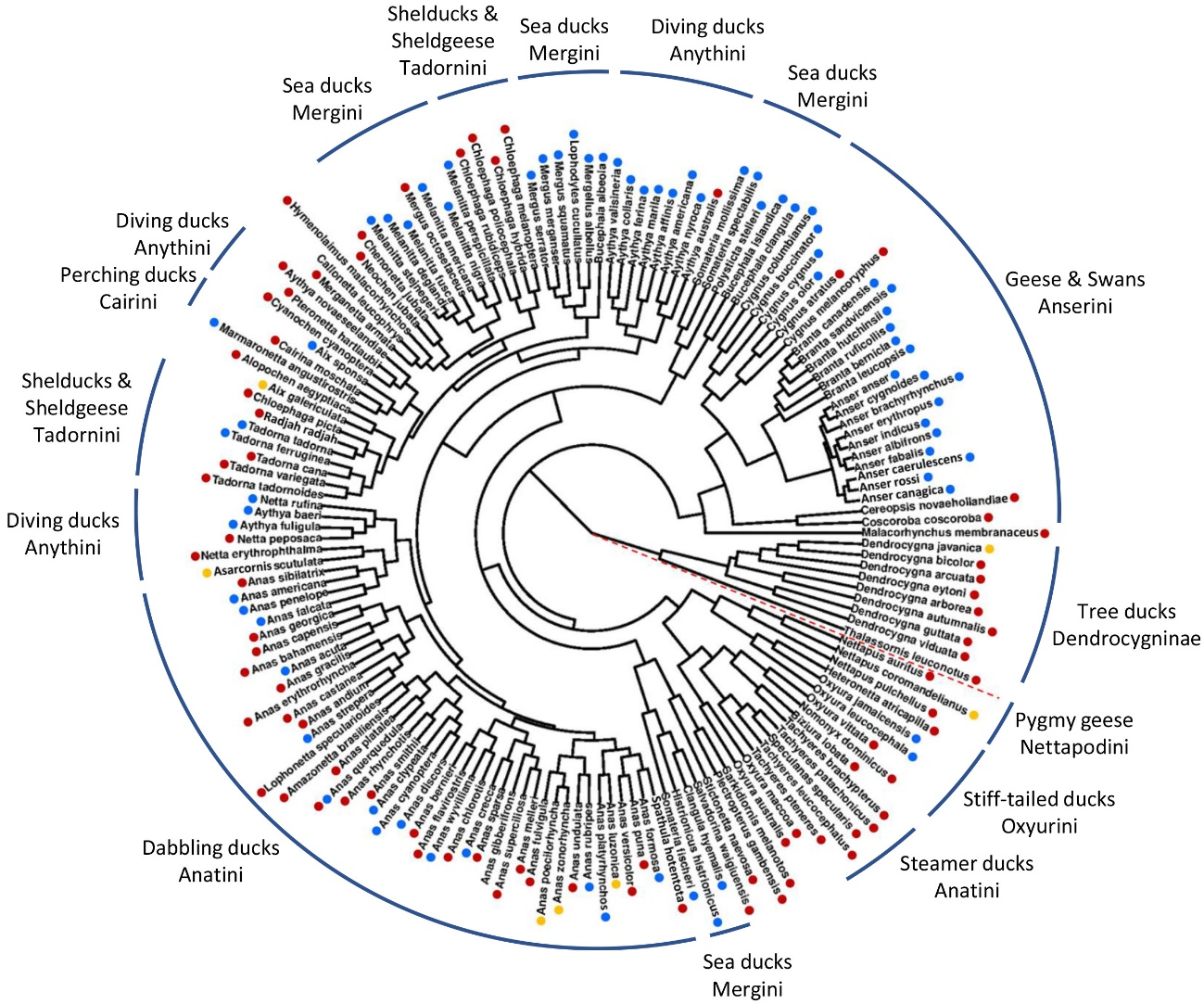

Supplement: Supplementary file 1 [file ECE3-9-9990-s001.tif]

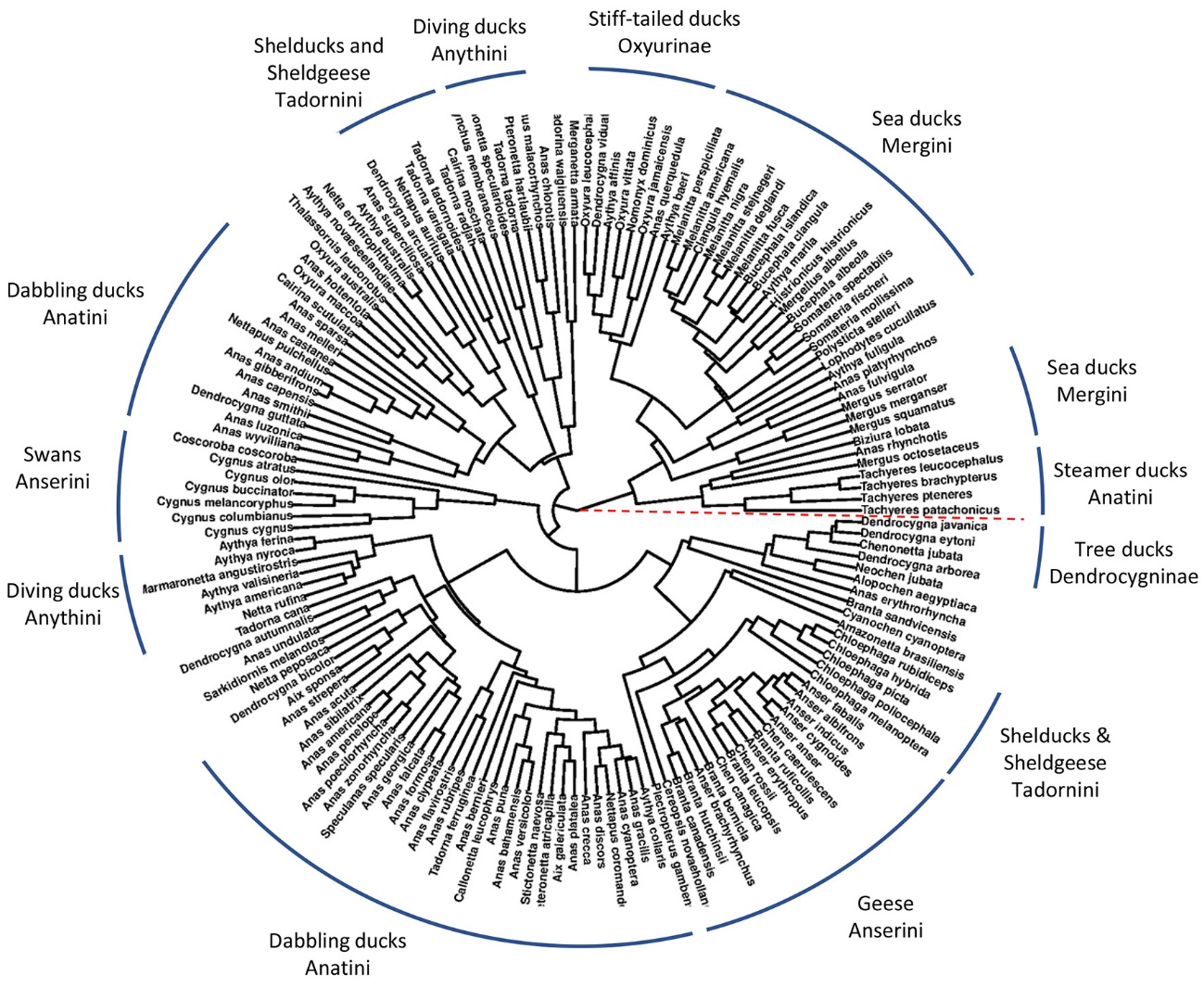

Supplement: Supplementary file 2 [file ECE3-9-9990-s002.tif]

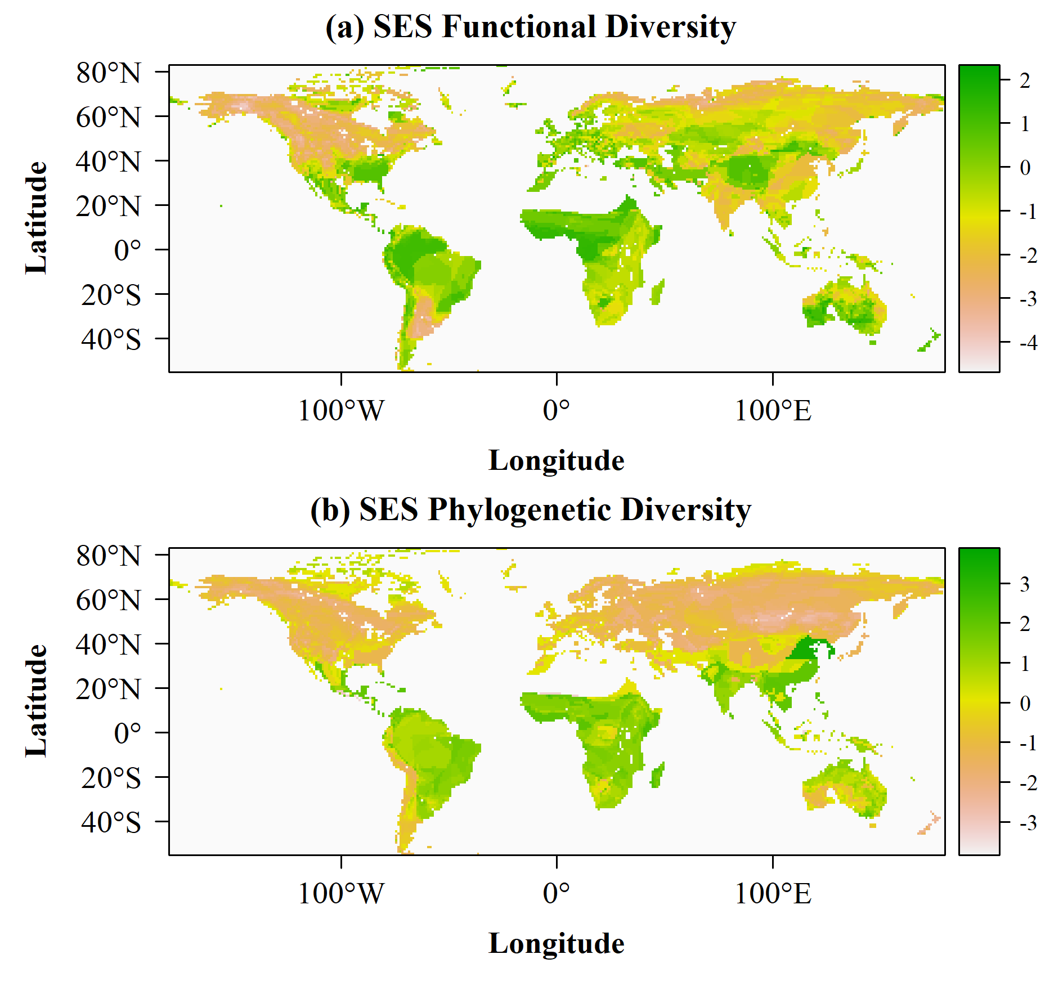

Supplement: Supplementary file 3 [file ECE3-9-9990-s003.tif]

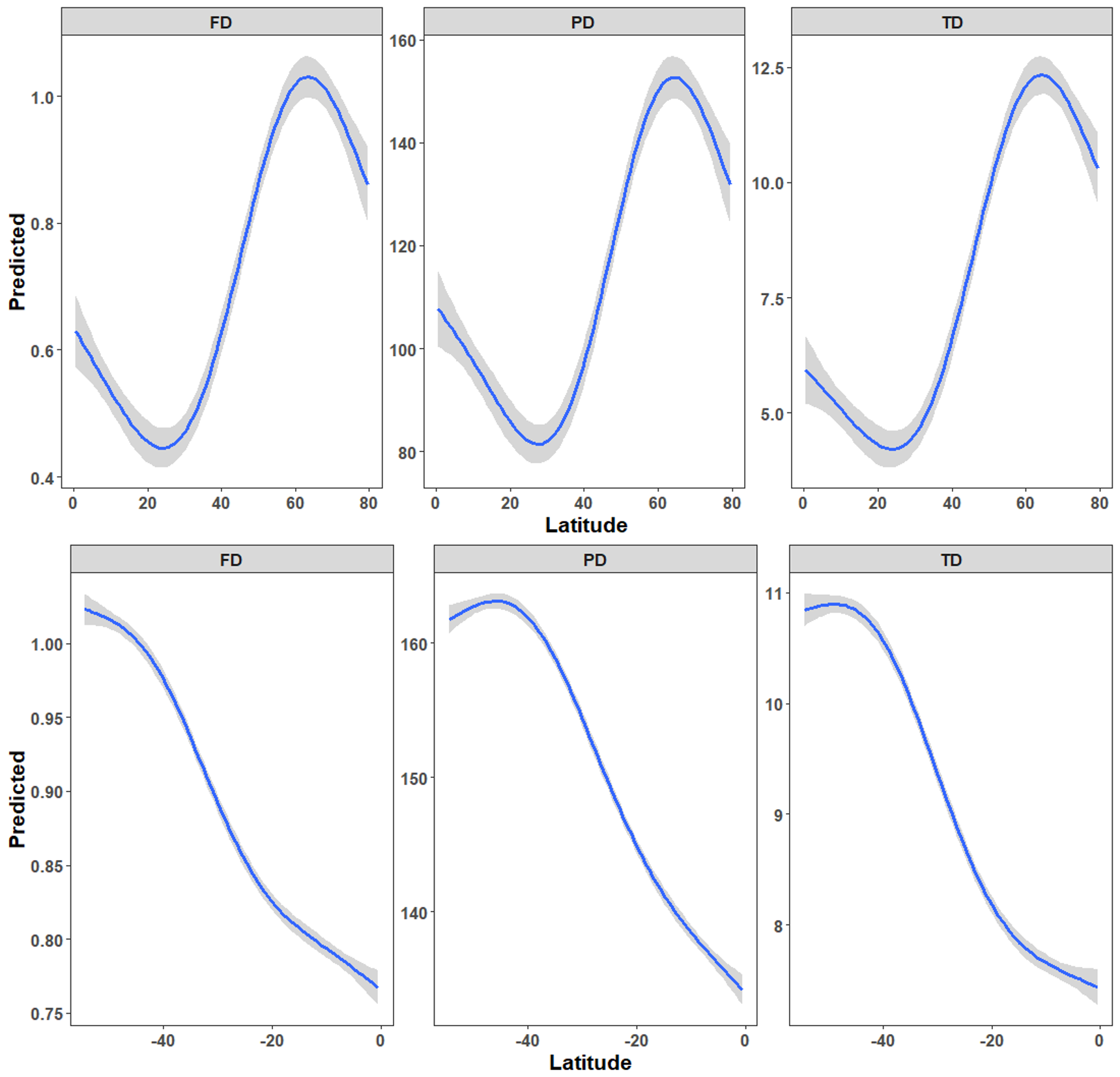

Supplement: Supplementary file 4 [file ECE3-9-9990-s004.tif]

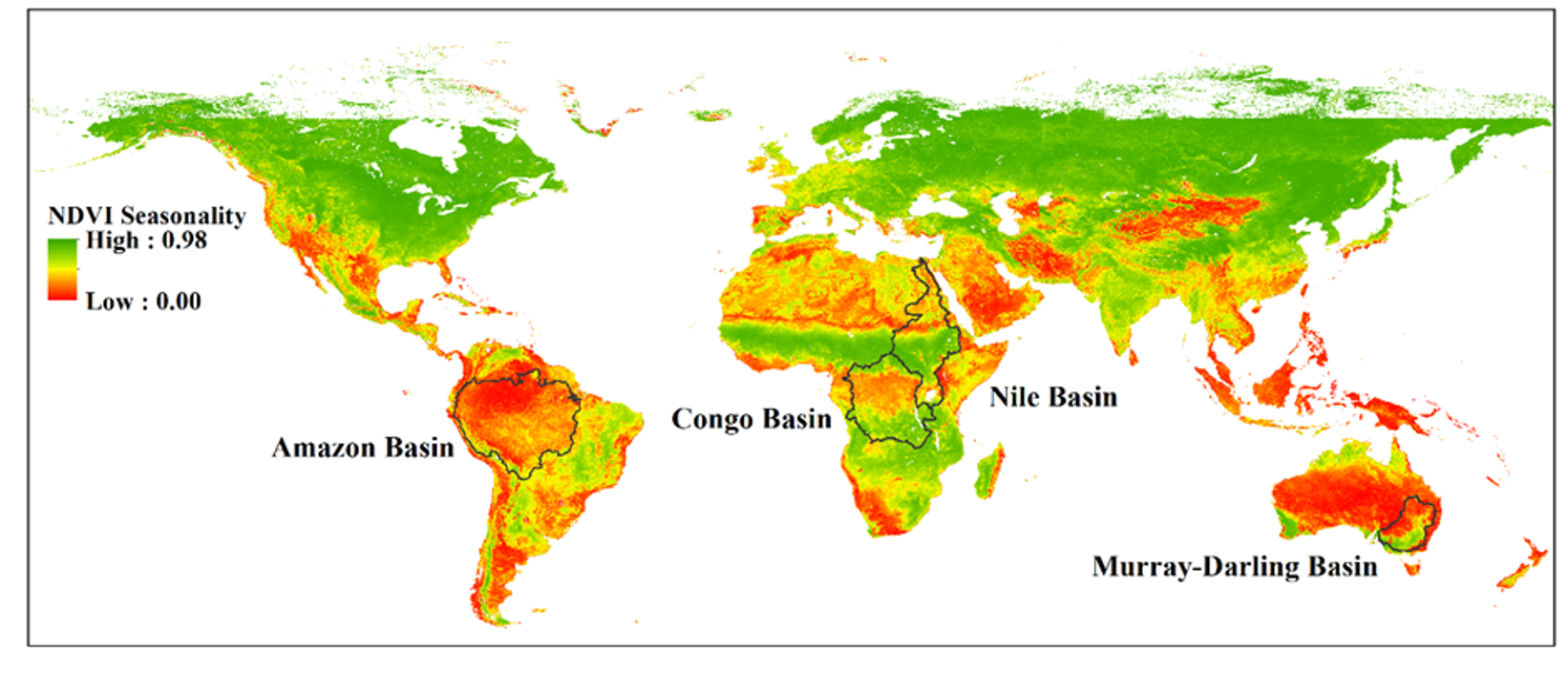

Supplement: Supplementary file 5 [file ECE3-9-9990-s005.tif]
