# Supplementary material for: Contrasting diversity patterns of breeding Anatidae in the Northern and Southern Hemispheres
Source: Ecol Evol. 2019 Aug 15;9(17):9990–10003. doi: 10.1002/ece3.5540 (PMC6746110; doi:10.1002/ece3.5540)
Supplement: Supplementary file 6 [file ECE3-9-9990-s006.docx]

Table S1 The 52 environmental variables used to characterise temporal and spatial environmental heterogeneity of 1° grid communities used to predict Anatidae diversity patterns

| **Category** | **Code** | **Description** |
| --- | --- | --- |
| Climate (38) | ^1^ mn.Bio01 - mn.Bio19 | Spatial average of the 30s resolution 19 Bio variables as defined by Hijmans et al. 2005 within each 1° x 1° cell. |
|  | ^2^ sd.Bio01 - sd.Bio19 | Climatic heterogeneity of the 19 Bio variables calculated as standard deviation of nine neighbour cells, and aggregated to 1° x 1° resolution. |
| Productivity (7) | mn.NDVI | Area averaged peak productivity , average of the 95^th^ percentile of 1 km resolution NDVI from 10-day composites between 2000 and 2009 (from <http://land.copernicus.eu/global/>) |
|  | season.NDVI | Average of annual NDVI seasonality (Coefficient of variation (COV) of the 36 10-day composites NDVI), aggregated to 1° resolution |
|  | het.NDVI | Spatial heterogeneity of NDVI, standard deviation of NDVI between the nine adjacent pixels, averaged within 1° x 1° window |
|  | annvar.NDVI | Standard deviation of annual peak productivity (defined as the 95^th^ percentile of 10-day composite NDVI) within 1° x 1° window |
|  | ^3^AET | Annual actual evapotranspiration from Global Soil Water Balance Geospatial Database aggregated to 1° x 1° resolution (<http://www.cgiar-csi.org>). |
|  | ^3^het.AET | Spatial variation of AET: standard deviation of AET within a 3×3 window and aggerated to 1° x 1° resolution. |
|  | ^3^season.AET | AET seasonality calculated as COV of monthly AET, resampled to 1° x 1° resolution |
| Hydrogeomorphology (7) | Distance.w | Spatial distance to inland waterbodies (data layer from GLWD (Global Lakes and Wetlands Database, <http://www.wwfus.org/science/data.cfm>). |
|  | ^3^ Lake.den | Density of lakes (% of grid cell) based on lake polygons from GLWD |
|  | Wetland.den | Percentage of wetland (data from GLWD) in the grid cell |
|  | Runoff | Average annual reliable runoff generated from the 1° cell based on 1961-1990, retrieved 24 June 2006 from GWSP Digital Water Atlas at <http://atlas.gwsp.org>. |
|  | sd.Elevation | Topographic heterogeneity calculated as standard deviation of nine neighbour cells, and aggregated to 1° x 1° resolution. |
|  | ^3^mn.Elevation | Mean elevation calculated using SRTM30 (30 second Shuttle Radar Topography Mission from <http://srtm.csi.cgiar.org>). |
|  | Irrigation | Irrigated land (km2) of each grid cell, data from World Water Development Report II <http://wwdrii.sr.unh.edu/download.html> |

^1^Seven of the 19 variables were selected based on variance inflation factor (VIF) analysis

^2^12 of the 19 variables were selected based on VIF analysis

^3^Not selected in the random forest modelling based on VIF analysis
